# Reporting the clinical spectrum of children with IgAV in a retrospective 24-year cohort: Influences of age and sex on clinical presentation

**DOI:** 10.55730/1300-0144.5700

**Published:** 2023-08-11

**Authors:** Sema YILDIRIM, Müferet ERGÜVEN

**Affiliations:** 1 Department of Pediatric, Göztepe Prof. Dr. Süleyman Yalçın City Hospital, İstanbul, Turkiye; 2 Department of Pediatric Rheumatology, Faculty of Medicine, Düzce University, Düzce, Turkiye

**Keywords:** Age, children, clinical manifestations, IgA vasculitis, sex

## Abstract

**Background/aim:**

Immunoglobulin A vasculitis (IgAV) is one of the most common types of vasculitis in children. The aims of this study were to investigate the clinical characteristics of the disease, and the effects of age and sex on the clinical course in children with IgAV.

**Materials and methods:**

This was a retrospective study including pediatric patients diagnosed with IgAV who attended follow-ups at the pediatric rheumatology department of a tertiary healthcare institution between January 1997 and December 2020. The patients were grouped and compared according to sex and age at diagnosis (<7 years vs. ≥7 years).

**Results:**

The study included 709 children with IgAV, 392 (55.3%) of whom were male. The mean age at diagnosis was 7.9 ± 3.2 years. The most common disease onset season was autumn (31.2%). Upper respiratory infections (27.8%) were the most common predisposing factors. Gastrointestinal system (GIS), joint, and renal involvement were observed in 52.8%, 47.5%, and 17.5% of patients, respectively. Renal involvement, GIS involvement, and disease relapse were significantly more common among those diagnosed after 7 years of age compared to those diagnosed before the age of 7 (p < 0.001, p = 0.033, and p < 0.001, respectively). Scrotal involvement and subcutaneous edema were more common among those diagnosed at younger than 7 years compared to those aged ≥7 years at diagnosis (p < 0.001 and p = 0.016, respectively). GIS involvement was more frequently seen in males compared to females (p = 0.046).

**Conclusion:**

It was demonstrated that being ≥7 years of age at diagnosis or being a male were associated with higher likelihood of renal and GIS involvement in children with IgAV.

## 1. Introduction

Immunoglobulin A vasculitis (IgAV), formerly named Henoch-Schönlein purpura (HSP), is one of the most common types of vasculitis in children. Histopathological findings of IgAV are characterized by the predominant deposition of IgA and/or immune complexes in small vessel walls [1, 2]. The disease is frequently seen in children, although it can occur at any age, even in adults. The presentation of IgAV in adults differs from that in children; adults rarely have abdominal pain and frequently have joint involvement [3, 4]. It is a typically benign self-limited illness and renal involvement is the most important factor affecting morbidity and mortality in children [3, 5]. However, in adults, renal involvement is frequently severe with poor outcome [6]. In previous study researchers have investigated predictor factors for system involvement such as the age of onset of disease, relapse of disease, atypical presentation of purpuric lesions, absolute neutrophil count, D-dimer, erythrocyte, sedimentation rate [7–11].

Clarifying the clinical progression and characteristics of patients throughout their childhood can provide important data that can be used to improve the management and follow-up of patients with IgAV.

In this study, the aim was to evaluate the clinical characteristics of IgAV in patients who were followed-up at the pediatric rheumatology department of a tertiary-level hospital in Türkiye. In addition, whether the clinical course of the disease is associated with age and sex was investigated.

## 2. Materials and methods

### 2.1. Study design

This study was performed by retrospectively evaluating pediatric patients diagnosed with IgAV who were followed-up at the pediatric rheumatology department of a single tertiary healthcare institution between January 1997 and December 2020.

The patients were grouped and compared according to sex and age at the time of diagnosis (<7 and ≥7 years). Patients who were followed-up for less than 6 months, those with missing relevant data, and those who presented with atypical skin lesions and had skin biopsy results that were inconclusive for IgAV were excluded from the study.

### 2.2. Data collection

Patient data were retrospectively reviewed from their medical files. The demographic data including age, sex, season on disease onset, history of infections, trauma, or surgeries within the month prior to disease onset, and clinical symptoms including skin, joint, and gastrointestinal system (GIS), or renal, scrotal, and central nervous system (CNS) involvement were evaluated. Moreover, imaging results such as abdominal ultrasonography (USG) and cranial magnetic resonance imaging (MRI), and the renal biopsy findings of the patients were recorded.

### 2.3. Definitions and disease manifestations

The patients were diagnosed with IgAV according to the European League Against Rheumatism, Pediatric Rheumatology International Trials Organization and Pediatric Rheumatology European Society (EULAR/PRINTO/PRES) criteria [12].

The time until diagnosis of the disease was defined as the time between the onset of symptoms and time of diagnosis.

GIS involvement was defined as the presence of abdominal pain, vomiting, occult blood in the stool, massive GIS hemorrhage and/or intussusception. Intussusception was detected using abdominal USG.

Renal involvement was defined as the presence of hematuria (>5 red blood cells per high-power microscopic field), proteinuria (spot urine protein/creatinine ratio >0.2 in children >2 years of age and 0.50 for infants aged 6–24 months or urine protein >4 mg/m^2^/h), hypertension (systolic and/or diastolic blood pressures ≥95th percentile), and/or renal failure. A urine protein/creatinine ratio of >2 or the presence of urine protein >40 mg/m^2^/h were used to define nephrotic proteinuria [12, 13]. Renal biopsy was performed in children who had severe initial findings of nephritic/nephrotic proteinuria, an increase in creatinine level, hypertension or oliguria, severe proteinuria that had existed for more than 4 weeks (spot urine U_a_/U_Cr_ >100 mg/mmol or >1 g/day/m^2^), mild proteinuria that had existed for more than 3 months (<1 g/day/m^2^), and impaired renal function. Histopathological analyses of IgAV nephritis (IgAVN) were evaluated using the International Study of Kidney Disease in Children (ISKDC) classification [14].

Persistence of IgAV was defined as disease that flared-up within 3 months of onset, and relapse was defined as disease that flared-up again after 3 months from onset.

Joint involvement was defined as arthralgia or arthritis based on physical examination. Scrotal involvement was evaluated based on a physical examination and confirmed using scrotal USG.

### 2.4. Statistical analyses

Statistical analyses were performed using IBM SPSS Statistics for Windows 25.0 (IBM, Armonk, NY, USA). The normal distribution assumption of the continuous variables was analyzed using the Kolmogorov-Smirnov test. Continuous data were expressed as the median (minimum–maximum) or mean ± standard deviation according to the normality of the distribution. Categorical variables were expressed as the absolute count (n) and percentage (%). Categorical variables were compared using the Pearson chi-squared test. P < 0.05 was considered statistically significant.

## 3. Results

This study included 709 patients, of which 392 (55.3%) were male. The mean age at diagnosis was 7.9 ± 3.2 (range: 1–17.8) years. The median time until diagnosis of the disease was 4 (1–90) days. The most common seasons the disease appeared in were autumn (31.2%) and spring (30.2%). Upper respiratory infections (27.8%), especially the common cold (18.3%), were the most common findings during this period (Table 1).

When the patients were investigated in regard to the first presenting symptom, purpura (77.9%), abdominal pain (12.7%), and arthritis (8.3%) were the most commonly observed. In 13.3% of the patients, purpura was accompanied by other clinical signs such as abdominal pain, arthralgia/arthritis, subcutaneous edema, and hematochezia as the initial clinical symptoms.

All of the patients were found to have purpura (100%) during the course of the disease. Moreover, 374 (52.8%) patients had GIS involvement, 337 (47.5%) had joint involvement, and 124 (17.5%) had renal involvement. Relapse of the disease was detected in 63 (8.9%) of the patients. The main clinical characteristics are shown in Table 2.

According to the age groups, 288 (40.6%) of the patients were <7 years old, while 421 (59.4%) were ≥7 years old. In the ≥7-year-old group, renal involvement (n = 94, 22.3%) was significantly more common compared to those younger than 7 years of age (n = 30, 10.4%) (p < 0.001). GIS involvement was also significantly more frequent in the ≥7-year-old group (n = 236, 56.1%) than in those younger than 7 years of age (n = 138, 47.9%) (p = 0.033). In addition, disease relapse was significantly more frequent in the ≥7-year-old group (n = 57, 13.5%) compared to the <7-year-old group (n = 6, 2.1%) (p < 0.001). However, scrotal involvement (among the males) was more common in children younger than 7 years of age (n = 21, 13%) compared to the ≥7-year-old group (n = 7, 3%) (p < 0.001), and subcutaneous edema was also recorded more frequently in the <7-year-old group compared to the ≥7-year-old group (p = 0.016). There were no significant differences between the groups in terms of joint involvement (p = 0.434) (Table 3).

When compared in regard to sex, GIS involvement was more frequently seen in the males (n = 220, 56.1%) compared to the females (n = 154, 48.6%) (p = 0.046). On the other hand, there were no significant differences between the males and females in terms of joint involvement (n = 146, 37.2% and n = 121, 38.2%; p = 0.800), renal involvement (n = 67, 17.1% and n = 57, 18%; p = 0.757), subcutaneous edema (n = 134, 34.2% and n = 98, 30.9%; p = 0.356), or disease relapse (n = 28, 7.1% and n = 35, 11%; p = 0.070) (Table 4).

## 4. Discussion

To our knowledge, this study presents data from the largest series of children with IgAV from a single center in Türkiye.

Peak incidence for IgAV presentation is between 4 and 6 years of age, and the great majority of cases are seen in patients younger than 10 years of age [2–4, 15]. In the current study, the mean age of disease onset was 7.9 ± 3.2 years. In a previous study, Calvino et al. reported the mean age as 6.2 ± 3.1 years [16]. In a recent study from Türkiye, the mean age of disease onset was reported as 7.5 ± 3.2 years by Karadağ et al. [17]. These results were quite similar to those in the current study. There are studies showing that IgAV predominantly presents in males [7, 8, 18]; however, others have found similar disease likelihood in females and males [17, 19]. Herein, it was found to present more in males than in females.

Although IgAV is the most common type of vasculitis in children, its etiology is still unclear. Previous epidemiological studies have found remarkable seasonal variations in HSP, with most cases occurring in the autumn and winter [20]. On the other hand, there are publications reporting that it is seen more frequently in the spring. While Wang et al. reported winter as the most common season for the onset of IgAV, Shim et al. observed that it was the spring [7, 9]. In their study, Hwang et al. reported that it was commonly seen in the spring and less in the summer [21]. Similar to the literature, in the present study, the autumn and spring were almost equal in terms of IgAV occurrence, and the summer was less common. Moreover, in another study from the same region of Türkiye, Karadağ et al. reported the spring as the most common season for IgAV onset and the summer as less common [17].

Other studies demonstrated the seasonal tendency with fewer cases seen during the summer months supporting the theory of viral precipitants triggering the onset of this disease [7, 9, 16, 17, 21]. In a recent study from South Korea that included over 16,000 children with IgAV, this was clearly demonstrated. The reesearchers looked at the seasonal variation of common viruses with IgAV incidence and found temporal relationships with respiratory syncytial virus, influenza, and norovirus [21]. Trapani et al. reported that 49% of patients had an infection as a potential triggering factor before IgAV onset. Furthermore, 42% of patients had respiratory tract infection, 5% had GIS infection, and 4% had other infections. However, 16% of patients had fever alone and 2% had vaccination/tick bite as a triggering factor [22]. Similarly, Calvino et al. found that 35.9% of patients had a history of upper respiratory tract (URT) infection before presenting with the disease [18]. Some studies have looked at the role of infectious foci located within the oral cavity and the ear, nose, and throat system [3]. In a cohort of children in Taiwan with IgAV, 36% were found to be positive for streptococci [23] Moreover, Calvino et al. and Salzbury reported that a positive throat culture was found in 20%–35% of patients with IgAV in their studies [13, 24]. Similar to previously published research, URT infection was also the most common triggering factor in the current study. Additionally, urinary tract infections, diarrhea, pneumonia, and hand-foot-mouth disease were other infections that were detected as triggering factors. The relatively less common triggers were vaccination, surgery, and trauma in the current study. Similarly, Trapani et al. found that 5% of patients had GIS infection and 4% had other infections, in addition to URT infection; however, 16% had fever alone and 2% had vaccination/tick bite as a triggering factor [22]. In a study from Italy, it was concluded that the measles-mumps-rubella vaccine was associated with a higher risk for the development of IgAV [25]. In a recent study, it was reported that SARS-CoV-2 vaccination might be a trigger for the development of IgAV by Hashizume at al. [26]. The detection of infections as the most common triggering factors supports the seasonal occurrence of IgAV. On the other hand, it may only indicate a normal course of life.

Skin involvement is present in all patients with IgAV, and the current study found that all of the patients developed purpura that was usually present with gastrointestinal pain and bleeding, kidney involvement, arthralgia, and/or arthritis [10, 16, 17]. In a previous study from Spain, Calvino et al. observed that 30.8% of children with IgAV had only skin manifestations, 38.4% had palpable purpura and nonskin manifestations (such as abdominal pain and joint manifestations), and 30.8% had only nonskin manifestation (abdominal pain or/and joint manifestations) as initial symptoms [16]. Chen et al. reported that GIS involvement occurred before the skin lesions were observed in 25.3% of patients [27]. Trapani et al. reported in their group of children with IgAV that purpura, joint, and GIS involvement were the presenting symptoms in 73%, 15%, and 12%, respectively [22]. The results herein were similar to the literature. Purpura may relapse in 25% of children with IgAV [2]. Relapse of the disease in the present patient group was less common.

Joint involvement including arthritis or arthralgia is present in 50% to 80% in children with IgAV [28]. Calvino et al. reported a joint involvement rate of 12.8%, while arthralgia was seen in 78.2% of children with IgAV [16]. Similarly, Trapani et al. reported that it was 74% in Italy. In 2 different studies from Türkiye, Çakıcı et al. reported that arthritis/arthralgia were observed in 42.4% in children with IgAV, and Karadağ et al. reported it as 54% [10,17]. The result herein for joint involvement was quite similar to other studies conducted in Türkiye. Karadağ et al. also found that there were no significant differences between male and female sex in terms of joint involvement [17]. Similarly, no significant differences were found between children <7 years of age and those aged 7 years, or male and female sex, in terms of joint involvement.

In the literature, GIS involvement that included abdominal pain, vomiting, diarrhea, occult bloody stool, hematochezia\melena, or intussusception occurred in 40% to 80% of children with IgAV [1, 7, 13, 27]. Wang et al. observed GIS involvement in 67.86% of their patients. Wang et al. reported 41.84% of patients had only abdominal pain and 26.02% had other symptoms, such as occult bloody stool or gross bloody stool [7]. In a study by Chen et al., GIS involvement was observed in 77.8% of patients, and 98.1% had abdominal pain, 39.5% had vomiting, 21.6% had GIS bleeding, and 6.8% had diarrhea. Moreover, 4 out of 208 patients with an atypical manifestation of IgAV were operated on due to the suspicion of acute appendicitis and peritonitis [27]. In a recent study, Karadağ et al. reported that GIS involvement was seen in 51.3% of patients, and 53.1% had abdominal pain, 6.4% had hematochezia, 4.2% had melena, 2.3% had intussusception, and 0.4% had pancreatitis or cholecystitis [17] Although the GIS involvement rate was similar to that reported by Karadağ et al., severe GIS involvement such as hematochezia/melena and intussusception were less common in the present study. Karadağ et al. observed that GIS bleeding was more common in males and severe GIS symptoms were more frequent in children >7 years of age [17]. Similarly, GIS involvement was present more frequently in males compared to females in the current study. Moreover, it was more common in children diagnosed after 7 years of age compared to those diagnosed before the age of 7.

Hematochezia and melena were present in 18 patients; 12 of whom were males and 11 were aged ≥7 years at diagnosis. Of these patients, 16 recovered with steroid therapy. Unfortunately, data concerning treatment were not accessible in the remaining 2 patients. Of the 10 patients with intussusception, 8 were male and 6 were ≥7 years of age at the time of diagnosis. Of the patients with intussusception, 3 required surgery, 1 patient was treated with pneumatic reduction, and 6 recovered with steroid therapy. According to these results, it may be possible to suggest that severe GIS complications are more common among males and patients diagnosed after 7 years of age. There was no relationship between disease relapse and GIS involvement.

Renal involvement was reported to be present in 20% to 61% of patients with IgAV in the literature [4, 6, 13, 19, 29]. The presenting symptoms of renal involvement range from microscopic hematuria to macroscopic hematuria, proteinuria, nephritic syndrome, nephrotic syndrome, hypertension, or renal failure [3, 4, 10, 13, 30]. It was opined herein that the huge variation with the previous studies regarding renal involvement rates may have been because those studies were conducted by different departments, such as pediatric nephrology, rheumatology, or pediatrics. In previous studies, researchers have reported that up to 30%–50% of children with IgAV develop hematuria and/or proteinuria in 4 to 6 weeks, and both were mostly mild and self-limited [16, 24, 31]. According to a study by Pohl, approximately 20% of IgAVN patients (7% of all IgAV cases) develop nephritic or nephrotic syndrome [3]. In tertiary centers, up to 20% of children with IgAV have progressed to chronic kidney disease, compared with less than 5% in unselected patients by 20 years after diagnosis [29]. Additionally, there are numerous studies which have defined risk factors for renal manifestation, including age, severe GIS manifestation, and disease relapse leading to IgAVN [7,32]. Çakıcı et al. reported that 37% of their patients had renal involvement, and being aged ≥8 years was a risk factor associated with the development of nephritis [10]. In another study from Türkiye, Demirtaş et al. reported renal involvement in 39.9% of patients with IGAV, and furthermore, they stated that GIS involvement and diastolic blood pressure were risk factors for IgAVN [31]. Wang et al. also reported renal involvement in 35.6% of their patients, 4.39% of whom had severe renal disease. In addition, among patients aged ≥6 years, severe renal involvement was found to be associated with CNS involvement and an interval >8 days between diagnosis and relapse [7]. In a metaanalysis study performed by Chan et al., male gender, an age >10 years, the presence of severe gastrointestinal symptoms (abdominal pain, gastrointestinal bleeding, and ischemic intestinal injury), persistent purpura, relapses of disease, and arthritis/arthralgia were reported as factors for developing nephropathy in the course of disease [33]. On the contrary, Salzbury reported that the incidence of nephritis was predominantly less common in children <2 years of age [24]. Renal involvement was observed more frequently in patients with disease relapse compared to those without, and also in patients diagnosed after the age of 7 compared to those diagnosed earlier (under 7 years of age). Severe renal involvement with nephrotic proteinuria was also more common among those aged ≥7 years. There was no obvious difference between the males and females in terms of renal involvement. Moreover, there was no correlation between renal involvement and the season of disease onset.

ISKDC scoring, which is based on the degree of mesangial proliferation and the percentage of glomeruli involving crescents, is recommended as the pathological classification of IgAVN in children [14]. Coppo et al. reported that most patients had class II and III ISKD in their study including 83 children and 136 adults with IgAVN [34]. According to the ISKDC classification, 2 patients had grade I, 1 had grade IIIa, 2 had grade IIIb, and 4 had grade II histological findings associated with IgAVN in the current study, similar to the literature. However, 2 patients did not have an ISKDC classification on their renal biopsy report. All of these patients were treated with steroids. None of the patients had end-stage renal disease. On the other hand, some previous studies reported that the severity of the first renal biopsy did not correlate with the risk of a poor outcome [35, 36].

While Çakıcı et al. reported the frequency of scrotal involvement at around 5.4%, Buscatti et al. and Ionnides et al. reported it as 19% and 30% in male patients with IgAV, respectively [10, 37, 38]. In the literature, some studies demonstrated the relationship between scrotal and renal involvement [39,40]. Kara et al. observed that 7 (14.8%) children with IgAVN had scrotal involvement; however, they did not find any significant correlation between scrotal involvement and renal outcome [41]. In the present study, the rate of scrotal involvement was closer to the result published by Çakıcı et al., and there was no relationship between scrotal and renal involvement. In addition, being >7 was detected as a risk factor for scrotal involvement.

Neurological symptoms such as headache, mental status changes, and seizures are rare clinical manifestations of IgAV [15]. Trapani et al. reported that 3% of patients had headache [22]. In another study, seizures were reported in 2% of patients [24]. In the present study, 1 patient had severe headache, a 14-year-old boy; 1 had diplopia, a 7-year-old boy; and both of their cranial MRIs were normal. Additionally, 1 patient, a 10-year-old boy, had seizures and his cranial MR imaging showed changes associated with posterior reversible encephalopathy in the bilateral parietooccipital and the frontal lobes. Moreover, the neurological findings found herein were exceedingly rare relative to the literature. The reason for this might have been that the most common neurological symptom such as nonspecific headaches, may not have been taken into consideration.

The main limitation of this study was its retrospective design. Data were collected from the patient medical files, which may have been recorded with varying levels of accuracy and/or details throughout the 24–year study period. However, the large number of patients and the long study period were important strengths of this study.

## 4. Conclusion

IgAV, which is the most type of common vasculitis in children, should be well known and managed by all pediatricians. The early determination of GIS and renal involvement are very important, as they are the most essential causes of morbidity in the disease. Although IgAVN has mostly a self-limiting and favorable resolution during the course of the disease, a subset of children, particularly those whose diagnosis and prompt intervention are delayed, are at risk of progression during the pediatric age or decades later into adulthood. In a previous study, the relation between renal involvement and age was mentioned; however, there is a void in the literature about GIS involvement and age. In this study, it was demonstrated that not only renal involvement, but also GIS involvement is more frequent in patients >7 years of age. Additionally, male gender is also an important risk factor in terms of GIS involvement in the course of the disease. Another important point that should be emphasized is that physicians should be more careful in terms of scrotal involvement in children <7 years of age.

## Figures and Tables

**Table 1:** Demographic characteristics of the patients with IgAV.

Sex (male/female), n (%)	**392/317 (55.3/44.7)**
Age (years), mean ± SD	**7.85 ± 3.25**
<7 years of age, n (%)	288 (40.6)
7 years of age, n (%)	421 (59.4)
Time until diagnosis median (min–max) (day)	**4 (1–90)**
Season of disease onset	**n (%)**
Autumn	**221 (31.2)**
Spring	**214 (30.2)**
Winter	**165 (23.3)**
Summer	**109 (15.4)**
Predisposing factor	**n (%)**
URT infection	**197 (27.8)**
Common cold	130 (18.3)
Tonsillitis	55 (7.8)
Sinusitis	8 (1.1)
Acute otitis media	4 (0.6)
Fever	**22 (3.1)**
Urinary tract infection	**11 (1.6)**
Diarrhea	**10 (1.4)**
Trauma	**6 (0.8)**
Others^a^	**21 (3)**
None	**442 (62.3)**

a Others: pneumonia, lymphadenitis, hand-foot-and-mouth disease, varicella, vaccine, appendectomy, insect bite, herpes labialis, hepatitis B, tooth abscess.

**Table 2:** Main clinical characteristics of the patients with IgAV.

First clinical sign^a^	n (%)
Purpura	**552 (77.9)**
Abdominal pain	**90 (12.7)**
Arthritis	**59 (8.3)**
Arthralgia	**45 (6.3)**
Subcutaneous edema	**39 (5.5)**
Myalgia	**8 (1.1**)
Vomiting	**7 (1)**
Hematochezia/melena	**4 (0.6**)
Testicular pain	**2 (0.3)**
Clinical signs	
Purpura (without thrombocytopenia)	**709 (100)**
GIS involvement	**374 (52.8)**
Abdominal pain	284 (40.1)
Occult stool blood	218 (30.7)
Vomiting	39 (5.5)
Hematochezia/melena	18 (2.5)
İntussusception	10 (1.4)
Joint involvement	**337 (47.5)**
Arthritis	257 (36.2)
Arthralgia	80 (11.3)
Renal involvement	**124 (17.5)**
Hematuria	80 (11.3)
Proteinuria	86 (12.1)
Nephritic	68 (9.6)
Nephrotic	18 (2.5)
High blood pressure	15 (2.1)
Renal biopsy supporting IgAVN	**11 (1.6)**
Subcutaneous edema	**232 (32.7)**
Scrotal involvement (n = 392 males)	**28 (7)**
Fever	**18 (2.5)**
Myalgia	**12 (1.7)**
CNS involvement	**3 (0.4)**
Relapse of disease	**63 (8.9)**
Persistent of disease	**108 (15.2)**

a Of the patients, 97 had more than 1 symptom as the first clinical sign at the same time.

GIS: gastrointestinal system, IgAVN: IgAV nephritis, CNS: central nervous system.

**Table 3:** Comparison of the clinical presentations of 709 children with IgAV in terms of age.

	Aged <7 years (n = 288) n (%)	Aged ≥7 years (n = 421) n (%)	*p-value
GIS involvement	138 (47.9)	236 (56.1)	0.033
Joint involvement	142 (49.3)	195 (46.3)	0.434
Renal involvement	30 (10.4)	94 (22.3)	<0.001
Subcutaneous edema	109 (37.8)	123 (29.2)	0.016
Scrotal involvement^a^	21 (13)	7 (3)	<0.001
Relapse of disease	6 (2.1)	57 (13.5)	<0.001

a only in male patients,

* p: chi-squared test.

**Table 4:** Comparison of the clinical presentations of 709 children with IgAV in terms of sex.

	Males (n = 392) n (%)	Females (n = 317) n (%)	*p-value
GIS involvement	220 (56.1)	154 (48.6)	0.046
Joint involvement	146 (37.2)	121 (38.2)	0.800
Renal involvement	67 (17.1)	57 (18)	0.757
Subcutaneous edema	134 (34.2)	98 (30.9)	0.356
Relapse of the disease	28 (7.1)	35 (11)	0.070

* p: chi-squared test.
